# The Correlation Analysis Between m6A Methylation Modification and Ferroptosis Induced by Cigarette Smoke in Human Bronchial Epithelium

**DOI:** 10.1002/iid3.70104

**Published:** 2024-12-17

**Authors:** Xiaomei Duan, Tingting Hu, Lijuan Xu, Zheng Li, Jing Jing, Dan Xu, Jianbing Ding, Fengsen Li, Min Jiang, Jing Wang

**Affiliations:** ^1^ Department of Xinjiang Laboratory of Respiratory Disease Research Traditional Chinese Medicine Hospital Affiliated to Xinjiang Medical University Urumqi Xinjiang China; ^2^ Department of Xinjiang Clinical Research Center for Respiratory Diseases Traditional Chinese Medicine Hospital Affiliated to Xinjiang Medical University Urumqi Xinjiang China; ^3^ Department of Endocrine Traditional Chinese Medicine Hospital Affiliated to Xinjiang Medical University Urumqi Xinjiang China; ^4^ Department of Immunology, College of Basic Medicine Xinjiang Medical University Urumqi Xinjiang China

**Keywords:** 16HBES, COPD, ferroptosis, N6‐methyladenosine methylation

## Abstract

**Background:**

Chronic obstructive pulmonary disease (COPD), a prevalent respiratory condition, is characterized by long‐term airway inflammation, which can lead to airway remodeling and persistent airflow restriction. Exposure to cigarette smoke is known as a major contributor to COPD development. Research has confirmed that ferroptosis and m6A modification are closely related to various inflammatory‐related diseases. However, the correlation between m6A methylation and ferroptosis in COPD has not been confirmed. In this study, combined with bioinformatics analysis and molecular biology methods we investigated how m6A methylation was correlated to ferroptosis‐associated genes (SLC7A11 and NQO‐1) in cigarette smoke induced 16HBES cells.

**Methods:**

Two microarray datasets (GSE30063 and GSE64614) were combined to identify differentially expressed genes (DEGs) through the application of bioinformatics techniques. A cigarette smoke (CS)‐induced 16HBE cells model was established. The ROS, GSH, MDA, and total iron content were detected by relevant detection kits. The expression levels associated with ferroptosis and m6A methylation modification‐related genes were determined via reverse transcription‐quantitative polymerase chain reaction and western blot.

**Results:**

Overall, 529 DEGs were identified in the above two databases. For COPD patients, significant changes were observed in FAGs (GCLC, NQO‐1, SLC7A11) and m6A methylation‐related genes (FTO). A negative correlation was also noted between the expression level of genes linked to ferroptosis (SLC7A11 and NQO‐1) and that of the m6A methylation gene (FTO). The in vitro experiments results indicate that SLC7A11 and NQO‐1 were significantly downregulated, and FTO were significantly upregulated. In addition, cigarette smoke stimulation increased the levels of MDA, LPO, and ROS, while reducing the content of GSH and total iron content in 16HBE cells.

**Conclusion:**

Our findings explored the relationship between ferroptosis and m6A methylation in COPD, and screened out SLC7A11, NQO‐1 and FTO may be critical in the pathogenesis of COPD.

## Graphical abstract

In this study, combined with bioinformatics analysis and molecular biology methods we investigated the correlation between N6‐methyladenosine (m6A) methylation and ferroptosis‐associated genes (SLC7A11 and NQO‐1) in 16HBES. Our research suggests that SLC7A11, NQO‐1, and FTO may be potential useful genes for identifying smokers at risk of COPD, and provide references and new ideas for the prevention and treatment of COPD as well as the research and development of future targeted drugs.

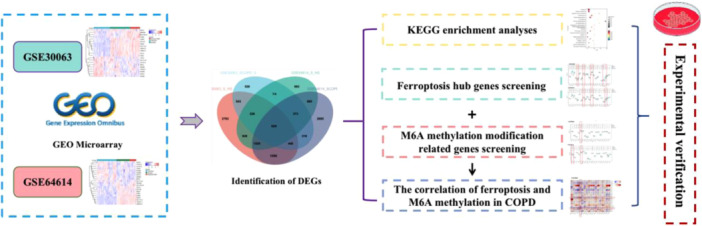

## 1 Introduction

Chronic obstructive pulmonary disease (COPD) is a prevalent chronic respiratory condition in which abnormalities in the airways and/or alveoli lead to enduring respiratory symptoms and airflow restrictions [1]. COPD ranks among the top four diseases worldwide in terms of incidence and mortality rates [2]. There are about 250 million patients who suffer from COPD [3]. Accounting for approximately 6% of total deaths in the world [4]. Currently, this disease has not yet been treated [4]. The abnormal inflammatory process caused by chronic smoking (CS) exposure is known to be partly responsible for the pathogenesis of COPD [2]. The harmful substances produced by CS could directly damage airway epithelial cells, leading to cell apoptosis and abnormal proliferation. This type of injury results in the airway epithelial cells losing their normal barrier function, rendering them vulnerable to attacks from external pathogens. Meanwhile, damaged epithelial cells also release various inflammatory mediators, thus intensifying the inflammatory response within the airways [5]. In addition, one of the pathological features of COPD is airway remodeling, CS could lead to airway wall thickening, smooth muscle proliferation, and fibrosis, among other airway remodeling phenomena. These alterations result in the narrowing of the airway lumen, limiting the airflow and consequently giving rise to symptoms including respiratory difficulties. Simultaneously, airway remodeling can also impact its compliance, rendering it more vulnerable to external stimuli. The harmful substances generated by smoking provoke immune cells in the airways, resulting in a persistent inflammatory reaction. This inflammatory response not only intensifies the damage and remodeling of the airways but also impairs the gas exchange function of the lungs, causing hypoxia and other manifestations, ultimately leading to cell death and other adverse consequences. In essence, the imbalance between airway epithelial cell proliferation and death homeostasis is the main cause of airway wall thickening and deformation [6]. Therefore, studying cell death patterns is of significant value to explore COPD's occurrence.

Ferroptosis, distinct from necrosis, autophagy, and apoptosis, is an emerging form of programmed cell death primarily marked by lipid peroxidation as well as the accumulation of iron intracellularly. These processes ultimately culminate in oxidative stress and subsequent cell demise [7]. The process of ferroptosis involves multiple signaling pathways and three major metabolic pathways (amino acid metabolism, iron metabolism and lipid metabolism) [8]. It has been established that ferroptosis could be important for the onset and evolution of various pathological processes and diseases, including ischaemic stroke [9], intracerebral hemorrhage [10], cancer [11], sepsis [12], and myocardial infarction [13]. It is believed that exposure to smoking causes an imbalance between oxidants and antioxidants, with the resulting oxidative stress subsequently leading to COPD. Exposure to CS‐containing oxidants can increase the burden of oxidants on the respiratory tract, enhance cell lysis and epithelial permeability, deplete antioxidant defense capabilities, and damage lung cells. Ferroptosis occurs when excessive lipid ROS cannot be removed by the antioxidant system consisting of NQO‐1, glutathione (GSH) and SLC7A11 [10]. Recent investigations have revealed the presence of ferroptosis‐related gene expression alterations in both COPD patients and mouse models, hinting at the possibility that ferroptosis could emerge as a promising therapeutic target for COPD [14], however, the potential mechanism is still unclear. Therefore, exploring the mechanism of ferroptosis in COPD airway epithelial cells, elucidating the crucial role of ferroptosis in the genesis and progression of COPD, and formulating effective COPD treatment strategies are of great significance.

N6‐methyladenosine (m6A) is a highly common modification that occurs after mRNA transcription. It is involved in regulating the localization, transcription, shear, and stability of mRNA [15, 16]. Smoking can cause a large amount of oxidative stress and inflammatory reactions in the body, which may affect the expression and activity of m6A‐modifying enzymes. Research has shown that smoking can alter m6A modification levels, thereby affecting gene expression regulation, especially in lung tissue [17]. In addition, smoking may also affect gene expression related to m6A modification, thereby affecting processes such as cell growth, proliferation, and apoptosis [18]. We conducted Me‐RIP sequencing using a COPD mouse model and found that m6A methylation modification can contribute to COPD's occurrence [19]. Therefore, our study searched for sequencing information of human bronchial epithelial cells induced by cigarette smoke through the Gene Expression Comprehensive Database (GEO), employed bioinformatics methods to predict the association between m6A methylation and genes linked to ferroptosis, and conducted in vitro validation through routine molecular biology experiments, to furnish a novel theoretical foundation for elucidating the pathogenesis of COPD.

## 2 Methods

### 2.1 Data Gathering and Pre‐Processing

This work used data available for airway epithelial samples from the GEO database, with accession numbers GSE64614 and GSE30063, respectively. The database GSE64614 includes 63 healthy nonsmokers, 73 healthy smokers, and 37 COPD‐S individuals, while the GSE30063 database includes 60 healthy nonsmokers, 73 healthy smokers, and 36 smokers with COPD. For detailed information on the two groups of database subjects, please refer to (GEO Accession viewer (nih.gov) and (GEO Accession viewer (nih.gov). The R package affyv1.78.0 was then used for normalizing all expression data.

### 2.2 Analysis of Differential Genes Expression

The Probe Set ID was converted into the gene symbol. If multiple probes were annotated to the same genes, the mean value was considered as the expression level of those genes. The Limma package in R software was then used for identifying differentially expressed genes (DEGs), with “*p* < 0.05” selected as the threshold for the screening.

### 2.3 KEGG Pathway Enrichment Analyses of Differential Genes

The cluster Profiler package (version 3.14.3) in R was used for KEGG pathway analysis, with the species limited to “*Homo sapiens*”, and results were considered as statistically significant at an “adjusted *p* value of < 0.05”.

### 2.4 Ferroptosis and m6A Methylation Modification

The Ferroptosis hub genes screening (FAGs) was derived from the systematic analysis by Tang et al. [20] The genes related to m6A methylation were obtained from the research conducted by Wang et al, [21] Wu et al, [22] and Qin et al [23] on the types, functions, and molecular characterization of m6A modulators. Correlations between m6A modification and ferroptosis across multiple genes were visualized using the pheatmap package. All the previously stated analytical techniques and R packages were put into practice by using the R Foundation for Statistical Computing (2020) version 4.0.3.

### 2.5 Validation of F**erroptosis Hub Genes**

#### 2.5.1 CSE Preparation

CSE was prepared as reported before [24]. After bubbling cigarette smoke through serum‐free Dulbecco's modified Eagle's medium (DMEM; 10 mL), the pH was adjusted to 7.35–7.45 using NaOH (1 mmol/L). Then, a 0.22 μm pore filter (Merck Millipore) was used to filter the DMEM (Gibco, cat.no.11965092) to remove bacteria and large particles before recording the absorbance values of the resulting liquid at 320 and 540 nm. Eventually, CSE solutions with ΔOD (A320–A540) between 0.9 and 1.2 were considered qualified and were regarded as a 100% concentration of CSE. In addition, the desired concentration of CSE was achieved by diluting it with a medium and it was subsequently utilized in experiments within 1 h.

#### 2.5.2 Cell Culture

The human bronchial epithelioid cells (16HBE) are an effective tool for simulating the exposure of respiratory epithelial cells to environmental factors such as smoking conditions. Consequently, the current study has procured 16HBE cells from the National Collection of Authenticated Cell Cultures to conduct subsequent in vitro experiments. The cells were cultured in DMEM medium containing 100 U/mL of penicillin, 100 μg/mL of streptomycin (Gibco, cat.no.15070‐063) as well as 10% fetal bovine serum (Gibco, cat.no.1248028) before incubation at 37°C with 5% CO_2_.

#### 2.5.3 Cell Viability Assay

Using a complete culture medium prepared 16HBE cells into a single‐cell suspension. The cell suspension was added to a 96‐well plate (Corning, cat.no.3599; 5 × 10^4^ cells/well), and following 24 h of CSE intervention, the medium was discarded from the well. Subsequently, 100 μL of a 10% CCK‐8 solution (TransGen Biotech, cat.no. FC101‐03) were dispensed into each well for a 1‐h incubation. Lastly, absorbance readings were taken at 450 nm using a microplate reader.

#### 2.5.4 Detection of Reactive Oxygen Species (ROS)

A Fluorometric Assay Kit (catalog number: E‐BC‐K138‐F) was employed, as required by the manufacturer, to determine ROS levels. Briefly, using a complete culture medium prepared 16HBE cells into single‐cell suspension (5 × 10^4^ cells/well). Adding 2 mL PBS and 2 μL DCFH‐DA (The final concentration is 10 μM) to each tube, incubate at 37°C for 30 min. Wash twice with PBS, and centrifuge collection of cell precipitates for flow cytometry detection.

#### 2.5.5 Detection of MDA and GSH

The 16HBE cells were first lysed using a cell lysis buffer (Beyotime, catalog number: P0013) through sonication, and after a 10‐min centrifugation at 700*g* and 4°C, the concentration of MDA and GSH in 16HBE cells was determined using MDA (ELabscience, cat.no.E‐BC‐K028‐M) and GSH (ELabscience, cat.no.E‐BC‐K030‐M) detection kits, with the results recorded at wavelengths of 532 and 450 nm, respectively.

#### 2.5.6 Measurement of Iron and Lipid Peroxide Levels

The overall iron concentrations present in cell lysates were measured using the Cell Total Iron Colorimetric Assay Kit (ELabscience, cat.no.E‐BC‐K880‐M). Briefly, the cell supernatants were collected after centrifugation. Add 80 μL of the iron probe and incubate at 37°C for 40 min. Absorbance readings were taken at 593 nm with a microplate reader. The lipid peroxide levels in cell lysates were assessed using lipid peroxide (LPO) colorimetric assay kit (ELabscience, cat.no.E‐BC‐K176‐M) adhering strictly to the manufacturer's instructions.

#### 2.5.7 Extraction of RNA and Quantitative Polymerase Chain Reaction

TRIzol Reagent (Ambion, 15596‐018) was used to isolate total RNA. To transcribe RNA into cDNA, 5X All‐In‐One RT MasterMix supplied by Abm (G492) was employed. RT‐PCR was carried out using EvaGreen Express 2× qPCR MasterMix (Abm, E824483) in conjunction with a Real‐Time PCR machine from ABI. The PCR protocol entailed an initial 10‐min heating step at 95°C, subsequently followed by 40 cycles, each involving 15 s of denaturation at 95°C and a 60‐s extension at 60°C for. The relative expression of the target gene was eventually determined using the $2^{‐∆∆C_{q}}$ approach, with normalization against GAPDH [25]. The primers used in this study (Table 1) were synthesized by BGI Biotechnology (Wuhan, China).

**Table 1:** The PCR primer sequences applied for RT‐qPCR.

Genes	Primer	Primer sequence (5′−3′)
*FTO*	Forward primer	ATTTCAGTCCATCATTCATGCG
	Reverse primer	GTAAAAACGTCCAACAGACCAA
*IGF2BP3*	Forward primer	TCTGCTACTTCCTCTACCAGAT
	Reverse primer	CAGGCCGAAATCACAAATCTTA
*WTAP*	Forward primer	ACACCACAGAAATCCCTAGAAG
	Reverse primer	CACAGCATCTGATAGAGAAGGT
*NQO1*	Forward primer	AGTATCCTGCCGAGTCTGTTCTGG
	Reverse primer	AATATCACAAGGTCTGCGGCTTCC
*SLC7A11*	Forward primer	TTGTTTTGCACCCTTTGACA
	Reverse primer	AAAGCTGGGATGAACAGTGG
*GAPDH*	Forward primer	GAGAAGGCTGGGGCTCATTTGC
	Reverse primer	TGCTGATGATCTTGAGGCTGTTGTC

### 2.6 Statistical Analysis

The statistical analysis of data derived from bioinformatics analyses was performed using R software (version 4.0.3). For in vitro experiments, results were compared in GraphPad Prism 8 software (GraphPad Software Inc., USA) using Student's *t*‐tests, with *p* < 0.05 selected as the threshold for significance.

## 3 Results

### 3.1 Identification of DEGs

**Figure 1:**
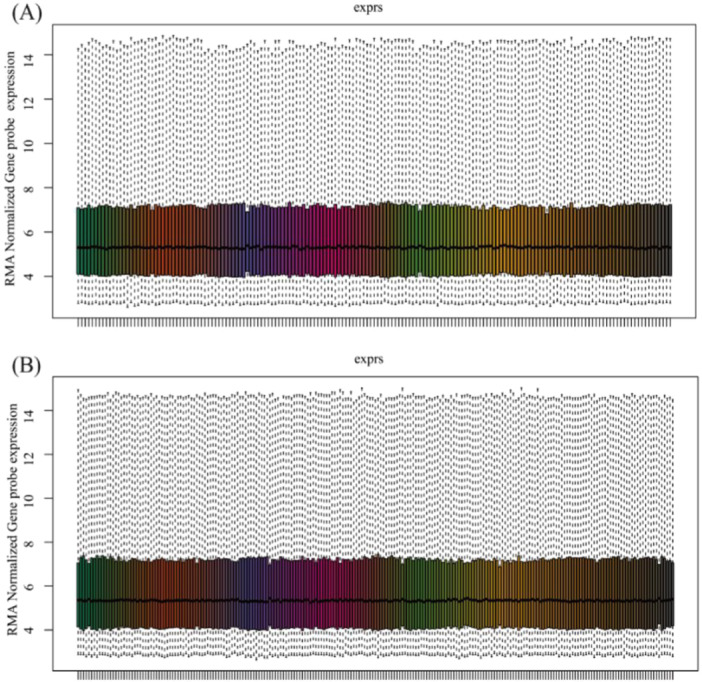
Normalized expression matrices of the datasets. Normalized expression matrices of the GSE30063 dataset (A). Normalized expression matrices of the GSE64614 dataset (B).

**Figure 2:**
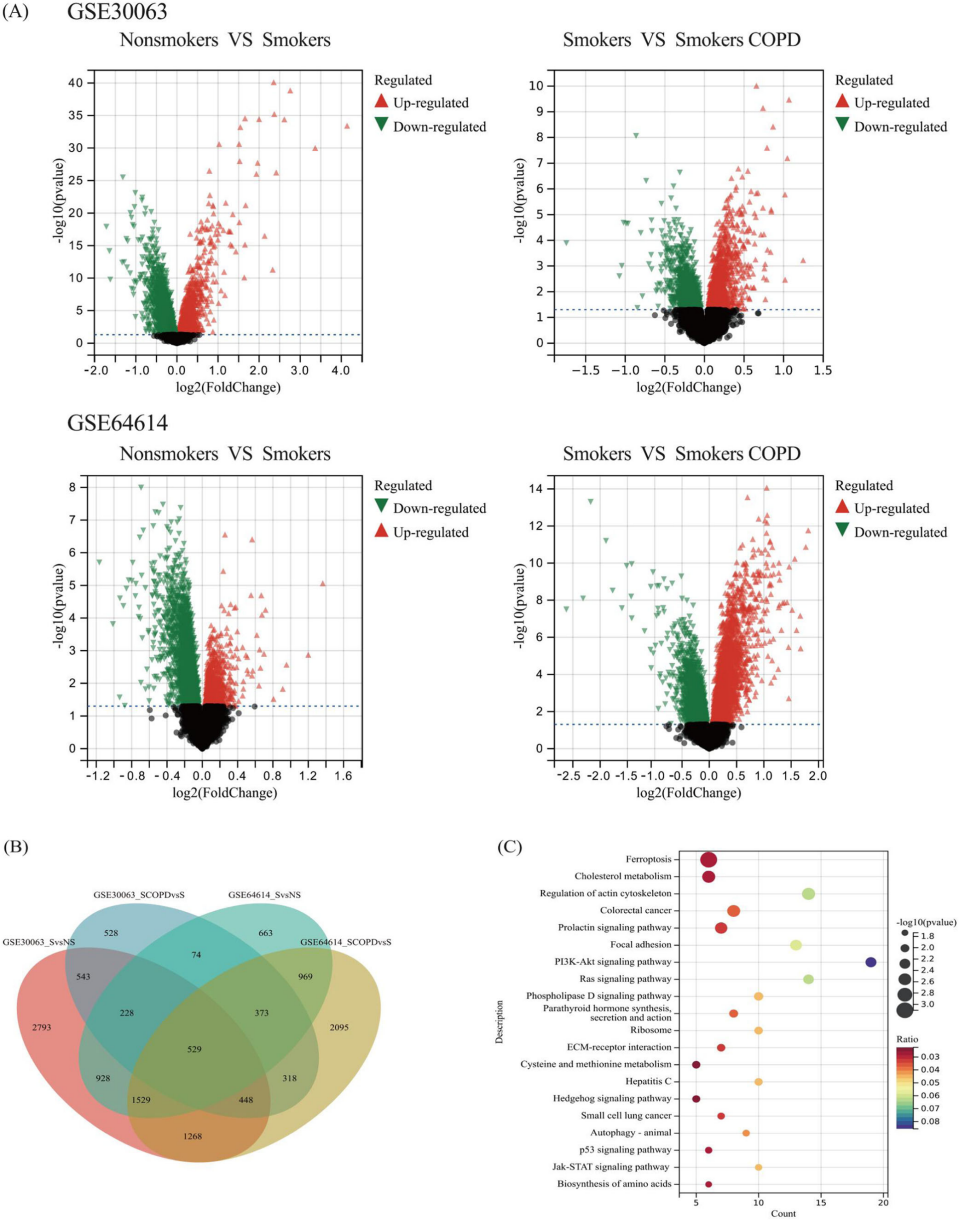
Differentially expressed genes of nonsmokers versus smokers and smokers versus smokers COPD of the GSE30063 and GSE64614 datasets. Presenting DEGs in the form of Volcano plots (A), Presenting DEGs in the form of Venn plots analysis (B), and the KEGG signaling pathways enrichment analysis result of DEGs (C).

Both datasets, GSE30063 and GSE64614, underwent normalization of their expression matrices and the box plots exhibited linear distribution trends as depicted in Figure 1A,B. Following the screening process using a threshold of an adjusted *p*‐value of < 0.05. When comparing nonsmokers and smokers in the GSE30063 dataset, a total of 8266 DEGs were discovered, of which the number of upregulated and downregulated genes were 3176 and 5090, respectively. Within the GSE64614 dataset, 5293 DEGs were recognized, including 1754 genes that showed upregulation and 3539 genes that demonstrated downregulation. In comparison between smoker and smoker COPD in the GSE30063 dataset, 3041 DEGs were detected, with 1780 and 1261 genes upregulated and downregulated, respectively. Analogously, the GSE64614 dataset revealed 7529 DEGs, of which 4521 were upregulated and 3008 were downregulated (Figure 2A). Figure 2B displays the constructed Venn diagrams, which demonstrate that a total of 529 genes showed differential expression across both datasets. To explore the underlying physiological functions of the 529 DEGs, R software was used for KEGG enrichment analysis. The enrichment histogram shows the top 20 results. As illustrated in Figure 2C, the analysis of the DEGs through the KEGG pathway indicated a remarkable enrichment in the ferroptosis pathway, cancer‐related signaling pathways, inflammation‐related signaling pathways, and metabolic‐related signaling pathways.

### 3.2 F**erroptosis Hub Genes Screening** (FAGs) in COPD Bronchial Epithelial Cells

**Figure 3:**
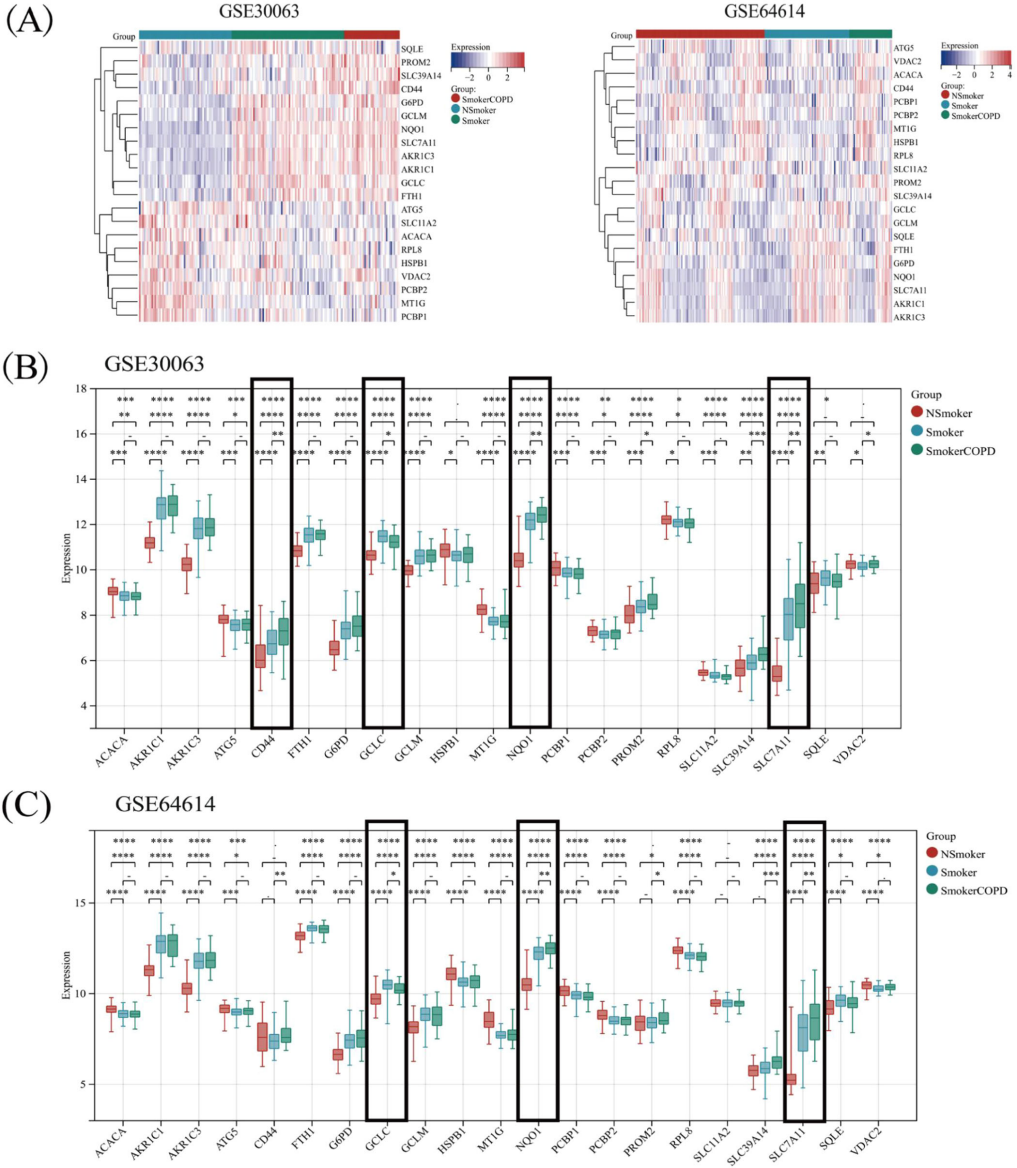
The expression distribution of ferroptosis‐related genes in COPD and control samples. The expression heatmap of ferroptosis‐related genes (A). Ferroptosis‐related genes analysis results by GSE30063 dataset (B). Ferroptosis‐related genes analysis results by GSE64614 datasets (C). *p < 0.05, **p < 0.01, ***p < 0.001, ****p < 0.0001.

According to previous literature, ferroptosis is closely related to various metabolic disorders including lipid metabolism, antioxidant metabolism, and iron metabolism [12]. Our study involved a comparison of the expression intensities of 529 DEGs in the bronchial epithelial cells among nonsmokers, smokers, and COPD smokers. In Figure 3A, ferroptosis‐associated gene expression patterns are displayed for both COPD and control specimens. ANOVA and Student's *t*‐tests were employed to compare the three groups and determine the results' significance. It was found that FAGs (GCLC, NQO‐1, SLC7A11) were significant differences between nonsmokers versus smokers and smokers versus COPD‐smokers in two datasets (Figure 3B,C). It is significant to observe that the increased expression of FAGs in COPD samples hints at ferroptosis's potential involvement in COPD development. Furthermore, when comparing nonsmokers with smokers, all of the ferroptosis‐related key genes depicted in Figure 3B exhibited notable alterations in the GSE30063 dataset, and in the GSE64614 dataset, significant changes were observed in all ferroptosis‐related genes except for CD44, PROM2, SLC11A2, and SLC39A14. The genetic alterations observed may provide insights into the underlying mechanism through which smoking triggers cell death. This offers an in‐depth understanding of how smoking can contribute to the development of various chronic diseases, including COPD.

### 3.3 Genes Exhibiting Correlation with m6A Methylation Modification

**Figure 4:**
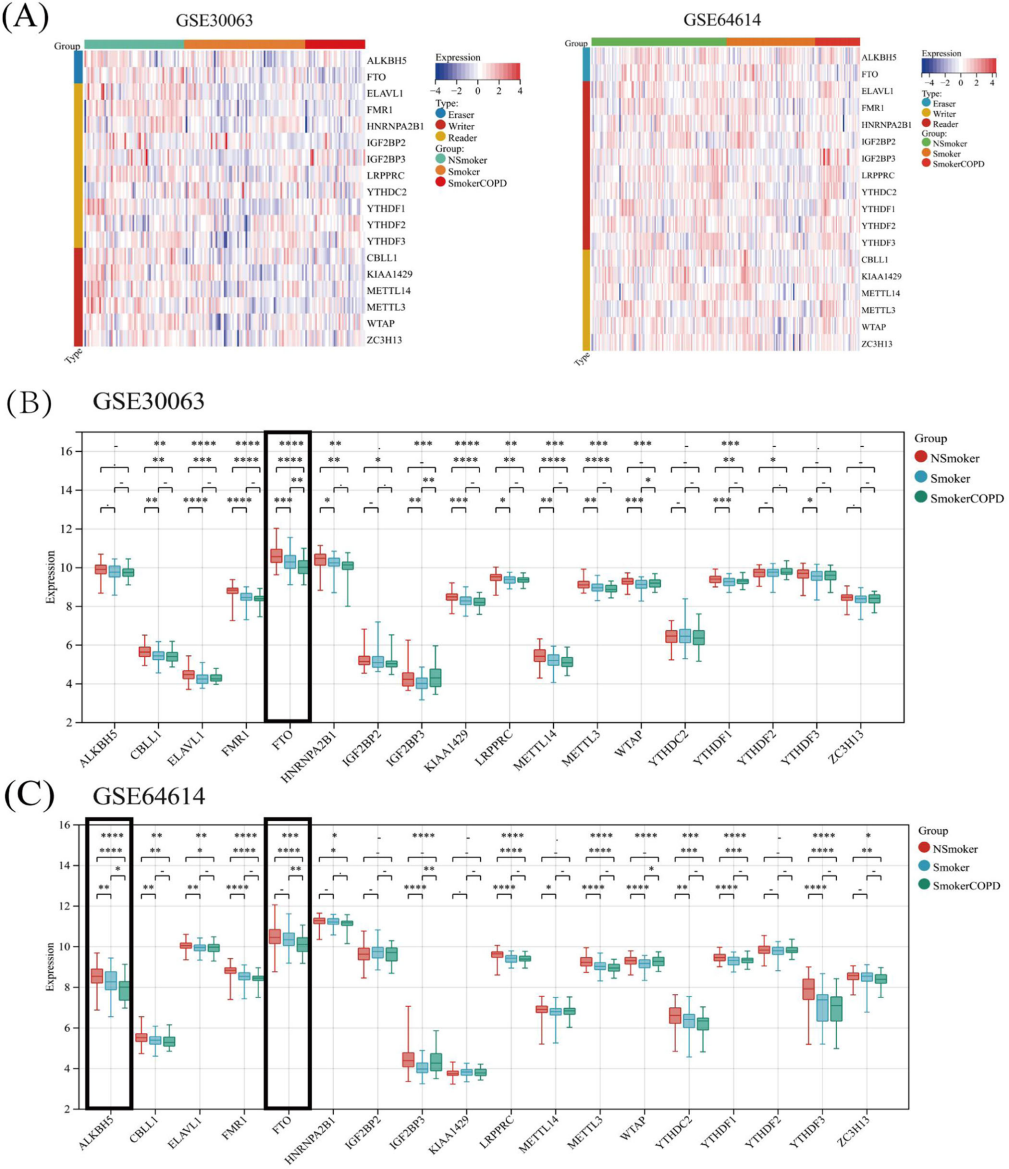
The expression distribution of m6A methylation modification‐related genes in COPD and control samples. The expression heatmap of m6A methylation modification‐related genes (A). m6A methylation modification‐related genes analysis results by GSE30063 dataset (B). m6A methylation modification‐related genes analysis results by GSE64614 datasets (C). *p < 0.05, **p < 0.01, ***p < 0.001, ****p < 0.0001.

RNA methylation encompasses three distinct categories of regulatory factors, comprising methyltransferases, demethylases, and RNA‐binding proteins [23]. This manuscript analyzed these three types of regulators in conjunction with the literature. Upon comparing nonsmokers with smokers, it was revealed that numerous genes involved in m6A methylation modification, including gene CBLL1, ELAVL1, YTHDF1, etc., underwent notable alterations. Compared with nonsmokers and Smokers, the expression of FTO diminished substantially in COPD patients in two datasets (Figure 4B,C). The expression profile of genes correlating with m6A methylation modification in both COPD and control samples is illustrated in Figure 4A. The information also pointed to the involvement of m6A methylation modification in COPD's onset and evolution.

### 3.4 The Association **B**etween Ferroptosis and m6A Methylation in COPD

The transcriptional activity of ferroptosis‐linked genes (NQO‐1, SLC7A11) and m6A methylation modification‐related genes (FTO) are all activated in COPD patients.

**Figure 5:**
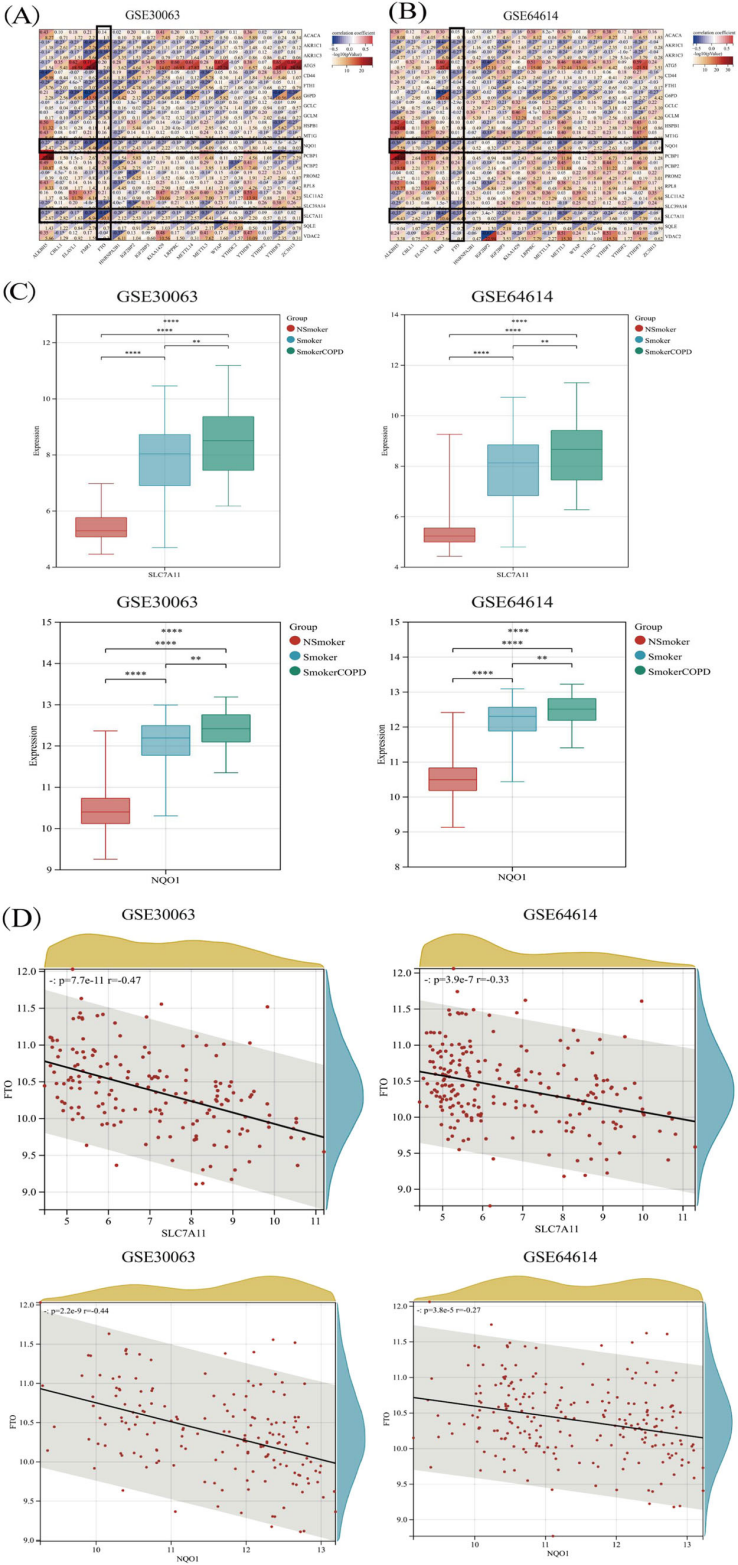
Correlation between ferroptosis‐related genes and m6A methylation modification‐related genes in COPD. Correlation between ferroptosis‐related genes and m6A methylation modification‐related genes in COPD by GSE30063 dataset (A). Correlation between ferroptosis‐related genes and m6A methylation modification‐related genes in COPD by GSE64614 dataset (B). Expression of ferroptosis‐related genes (NQO‐1, SLC7A11) in COPD patients (C). Scatter diagram of correlation analysis between key ferroptosis genes (NQO‐1 and SLC7A11) and key M6A methylation modification‐related genes (FTO) (D).

Nonetheless, the association between these genes has yet to be further examined. A significant negative linkage between the expression levels of FAGs (NQO‐1, SLC7A11) and the m6A methylation gene FTO was observed during Pearson's correlation analysis, with the results presented in Figure 5A,B,D. In addition, in smoking COPD patients, the expression of ferroptosis‐related genes (NQO‐1, SLC7A11) increased significantly (Figure 5C), therefore, we believe that NQO‐1 and SLC7A11 may be worthy of further study.

### 3.5 Validation of Ferroptosis Hub Genes

**Figure 6:**
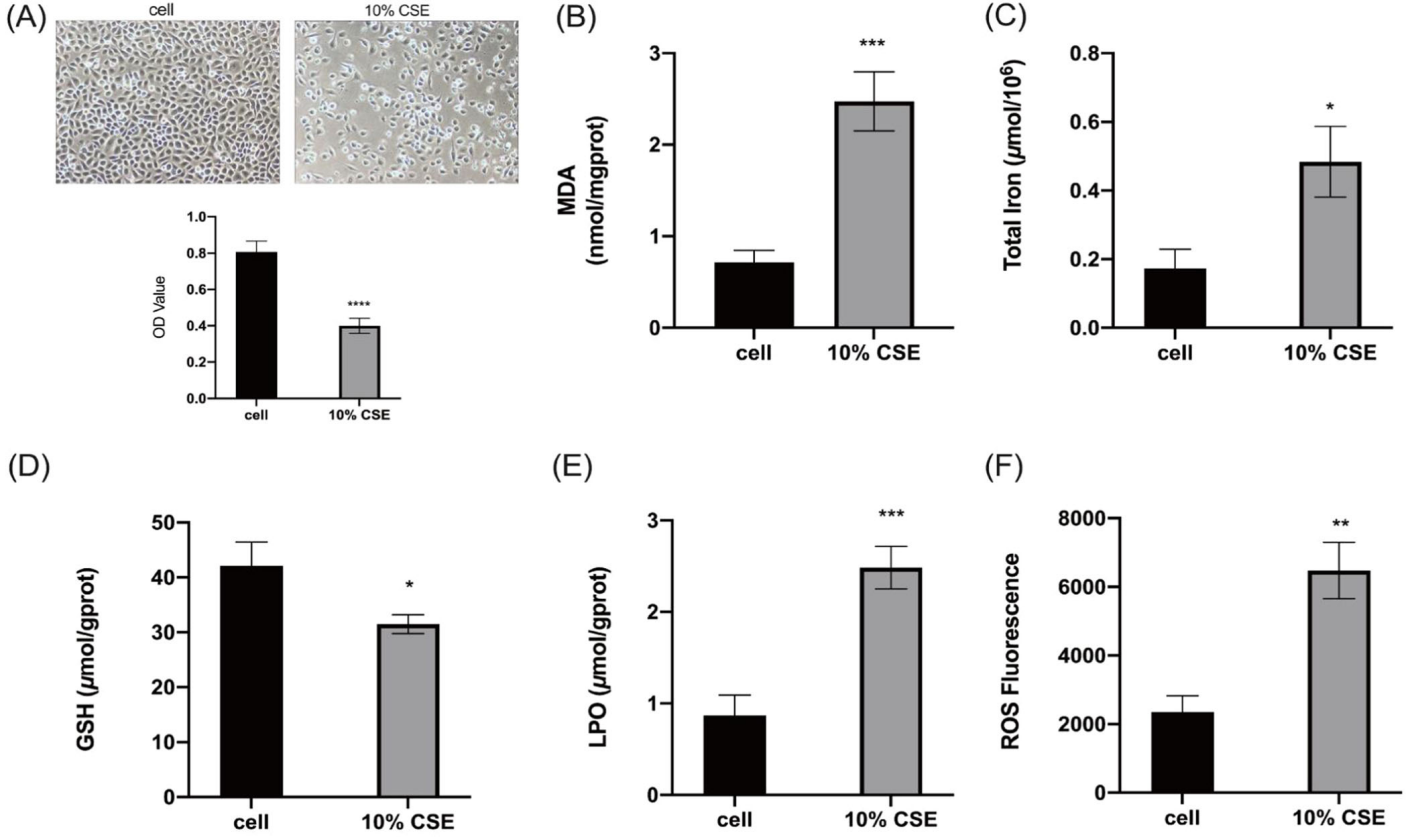
Cigarette smoke induces ferroptosis in bronchial epithelial cells. The effect of 10% CSE on the viability of 16HBE cells (A). MDA levels in cigarette smoke‐induced 16HBE cells (B). Total iron levels in cigarette smoke‐induced 16HBE cells (C). GSH levels in cigarette smoke‐induced 16HBE cells (D). LPO levels in cigarette smoke‐induced 16HBE cells (E). Relative levels of ROS in cigarette smoke‐induced 16HBE cells (F). *p < 0.05, **p < 0.01, ***p < 0.001.

**Figure 7:**
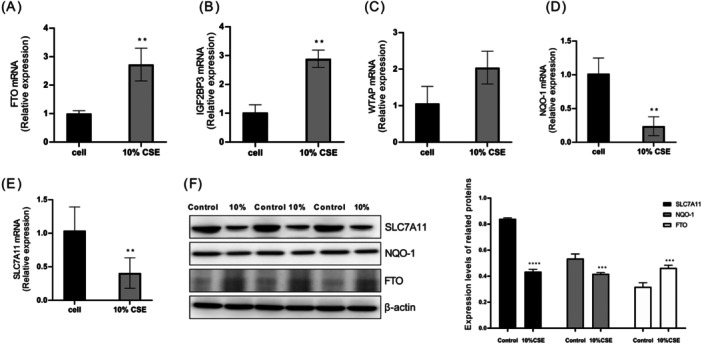
Validation of key ferroptosis genes and m6A methylation modification related factors expression changes in cigarette smoke‐induced bronchial epithelial cells by RT‐qPCR and Western blot. Changes in mRNA expression of FTO (A), IGF2BP3 (B), WTAP (C), NQO‐1 (D), and SLC7A11 (E) and protein expression of FTO, NQO‐1 and SLC7A11 (F) in 16HBE cells induced by cigarette smoke. *p < 0.05, **p < 0.01, ***p < 0.001.

Defined by the excessive buildup of ROS and iron‐reliant lipid peroxidation, ferroptosis represents a new type of necrotic cell death [26]. Therefore, this manuscript used a DCFH‐DA probe to measure the ROS content, and the MDA, GSH, and LPO detection kits were used to detect the relevant indicator levels in 16HBE cells. CCK‐8 assay for detecting cell activity. Based on the results, CSE pretreatment led to a significant increase in the MDA, LPO, ROS, and total iron levels (Figure 6B,C,E,F), at the same time, the GSH content was reduced (Figure 6D). In addition, the cell activity of 16HBE cells were significantly decreased after stimulation by 10% CSE (Figure 6A). Above experimental results showed that cigarette smoke could cause ferroptosis in 16HBE cells. Bioinformatics analysis results show that m6A methylation modification‐related genes (FTO) and ferroptosis‐related genes (NQO‐1, SLC7A11) are all activated in COPD patients. Our manuscript determined the above targets by RT‐qPCR and western blot. The experimental results indicated that m6A methylation modification‐related factors FTO and IGF2BP3 were significantly upregulated after 10% CSE induction (Figure 7A,B). Although WTAP increased after 10% CSE stimulation, there was no statistical difference (Figure 7C). At the same time, ferroptosis‐related factors NQO‐1 and SLC7A11 were significantly downregulated in cigarette smoke‐induced 16HBE cells (Figure 7D,E). Our results have proved that m6A methylation modification and ferroptosis were closely linked to COPD, Additional work on the specific regulation mechanism to clarify the correlation between FTO and NQO‐1, and SLC7A11 is our further research plan. Targeting m6A and ferroptosis‐related genes could potentially result in an alternative approach for treating COPD.

## 4 Discussion

COPD is a disease characterized by abnormal airway inflammation, which can lead to structural changes in the airways, vessels, and lung parenchyma [26]. By 2030, it is anticipated that this condition will become the third most frequent cause of death [27]. Hence, recent research has been increasingly focused on diagnosing and treating COPD, but given our restricted comprehension of the disease's pathogenesis as well as the lack of specific drugs, COPD patients still have poor prognosis [14]. Smoking poses a significant risk of developing COPD. As a result, it is vital to explore further the genetic differences and potential pathogenic mechanisms that exist between smokers who remain healthy and those who develop COPD. Currently, multiple molecular mechanisms have been proposed to induce COPD. Among them, ROS and LPS‐induced oxidative damage and inflammation in the lungs are considered key mechanisms of COPD. Moreover, the latest research indicates that ferroptosis represents a potential target for treating COPD. Exposing in vitro and in vivo models to cigarette smoke has also been shown to enhance lipid peroxidation and cause unstable iron accumulation, thereby resulting in ferroptosis [5, 28], and the protection of ferroptosis inhibitor ferrostatin‐1 in COPD models conclusively demonstrated the participation of ferroptosis in the occurrence of COPD [29]. Our findings indicate that ferroptosis is a vital aspect of COPD. Our results also confirm that the lung tissues of COPD patients exhibit significant changes in the key proteins of ferroptosis (NQO‐1 and SLC7A11). SLC7A11 and NQO‐1, as key ferroptosis genes, are crucial factors in COPD's pathogenesis. Hence, a close link is observed between changes in their expression level and disease progression as well as the severity of COPD. Therefore, these genes have great potential as molecular markers of COPD. By monitoring the expression of SLC7A11 and NQO‐1, we can identify the risk of COPD earlier, evaluate disease progression, and provide individualized treatment plans to patients. This discovery provides new ideas and methods for diagnosing and treating COPD, and is expected to make important contributions to improving the prognosis and quality of life of COPD patients.

Previous investigations have shown that factors regulating m6A RNA methylation may be responsible for causing COPD [30, 31]. However, current evidence is inadequate to establish a relationship between ferroptosis and m6A modification in COPD models. Therefore, our study conducted a preliminary exploration of the above correlation. The bioinformatics analysis results showed that the differential expressed genes of ferroptosis (NQO‐1 and SLC7A11) have a significant negative correlation with the differential expressed genes of m6A (FTO). Despite that, our study was not without restrictions. Firstly, the bioinformatics methods used statistical and sequencing results from others which may not have been free from false positive or false negative errors. The GSE30063 and GSE64614 datasets respectively contained 36 and 37 COPD patients, while the number of healthy controls were 133 and 136 healthy, respectively. Hence, the restricted sample size might contribute to a rise in the false‐positive rate. We speculate that it is precisely for this reason that the trends predicted by bioinformatics are different from the results verified by our in vitro experiments and Me‐RIP sequencing (Data not displayed) [17]. In addition, after searching the GEO database for bronchial epithelium stimulated by cigarette smoke, it was found that most of the databases searched were from institution Weill Cornell Medical College, Department of Genetic Medicine. The above two points may be the reasons why the bioinformatics predicted trends do not match the actual detected trends (Bioinformatics predicted that NQO‐1 and SLC7A11 were upregulated after CSE stimulation, while RT‐qPCR, WB and Me‐RIP sequencing results showed a decrease in the expression of these two genes following CSE stimulation). Secondly, despite discussing the association between m6A methylation and ferroptosis utilizing bioinformatics, we were unable to determine the causal link between the two as experimental confirmation was absent. Thirdly, gene expression in animal and human tissues has not been further experimentally verified. In the following research, it is necessary to improve the collection and testing of animal and clinical data as much as possible, while clarifying the role of specific genes in COPD through mechanism research.

In conclusion, we identified 529 DEGs by GSE30063 and GSE64614 datasets. Potential ferroptosis genes (NQO1 and SLC7A11) and m6A methylation‐related genes (FTO) showed good diagnostic characteristics. In addition, the analysis of these hub genes revealed that FAGs (SLC7A11 and NQO‐1) were negatively linked to the expression levels of the m6A methylation genes (FTO) (*p* < 0.05). In vitro experiments have also effectively verified the predicted results of bioinformatics. The discoveries as mentioned above enhance our comprehension of the distinct association between ferroptosis and COPD, suggesting that a thorough investigation of m6A methylation modification has the potential to yield therapeutic targets for COPD sufferers.

## Author Contributions

**Xiaomei Duan:** conceptualization, initial writing draft, writing revision, and editing. **Tingting Hu:** conceptualization, initial writing draft, writing revision, and editing. **Lijuan Xu:** data curation, formal analysis, resources, software. **Zheng Li:** data curation, formal analysis, resources, software. **Jing Jing:** investigation, supervision, validation. **Dan Xu:** investigation; methodology, visualization. **Jianbing Ding:** software, supervision. **Fengsen Li:** methodology, validation. **Min Jiang:** project administration, supervision. **Jing Wang:** project administration, supervision.

## Ethics Statement

The Ethics Committee of Xinjiang Uygur Autonomous Region Chinese Medicine Hospital approved this study (IACUC‐20240521‐26).

## Conflicts of Interest

The authors declare no conflicts of interest.

## Data Availability

The datasets used or examined in this work are obtainable from the corresponding author upon receipt of a legitimate request.
